# HIGHER COPING SELF-EFFICACY ASSOCIATED WITH LOW SELF-PERCEIVED LONELINESS IN OLDER ADULTS WITH CHRONIC DISEASE

**DOI:** 10.1093/geroni/igz038.228

**Published:** 2019-11-08

**Authors:** Melissa D Hladek, Paula V Nersesian, Thomas K Cudjoe, Jessica M Gill, Sarah L Szanton

**Affiliations:** 1 Johns Hopkins University School of Nursing, Baltimore, Maryland, United States; 2 Johns Hopkins University School of Medicine, Baltimore, Maryland, United States; 3 NIH, NINR, Bethesda, Maryland, United States

## Abstract

Loneliness is an emotional state involving social network perceptions and linked to worse health outcomes. Coping self-efficacy evaluates confidence in ability to manage problems effectively using problem-solving, emotional regulation and social coping. The purpose of this cross-sectional study (N=151 community dwelling adults ages ≥ 65) was to evaluate associations between loneliness and coping self-efficacy. All participants had at least one chronic condition and were cognitively intact. In this sample, 32.08% were lonely (score ≥ 5 on UCLA 3-item loneliness scale (range 3-9). Higher coping self-efficacy was significantly associated with low loneliness after adjustment for age, sex, race/ethnicity, social support, depressive symptoms, body mass index, and a chronic disease-function score (β= -0.03, p=0.014). Causality could not be assessed; higher loneliness may lead to lower self-efficacy or lower self-efficacy may lead to higher loneliness. Nonetheless, loneliness and self-efficacy are both modifiable with great potential for improvement, possibly bettering health outcomes.

